# Local environmental quality positively predicts breastfeeding in the UK’s Millennium Cohort Study

**DOI:** 10.1093/emph/eox011

**Published:** 2017-08-21

**Authors:** Laura J Brown, Rebecca Sear

**Affiliations:** Department of Population Health, London School of Hygiene & Tropical Medicine, Keppel Street, London WC1E 7HT, UK

**Keywords:** breastfeeding, maternal investment, environmental quality; life history theory; SES; perception

## Abstract

**Background and Objectives:** Breastfeeding is an important form of parental investment with clear health benefits. Despite this, rates remain low in the UK; understanding variation can therefore help improve interventions. Life history theory suggests that environmental quality may pattern maternal investment, including breastfeeding. We analyse a nationally representative dataset to test two predictions: (i) higher local environmental quality predicts higher likelihood of breastfeeding initiation and longer duration; (ii) higher socioeconomic status (SES) provides a buffer against the adverse influences of low local environmental quality.

**Methodology:** We ran factor analysis on a wide range of local-level environmental variables. Two summary measures of local environmental quality were generated by this analysis—one ‘objective’ (based on an independent assessor’s neighbourhood scores) and one ‘subjective’ (based on respondent’s scores). We used mixed-effects regression techniques to test our hypotheses.

**Results:** Higher objective, but not subjective, local environmental quality predicts higher likelihood of starting and maintaining breastfeeding over and above individual SES and area-level measures of environmental quality. Higher individual SES is protective, with women from high-income households having relatively high breastfeeding initiation rates and those with high status jobs being more likely to maintain breastfeeding, even in poor environmental conditions.

**Conclusions and Implications:** Environmental quality is often vaguely measured; here we present a thorough investigation of environmental quality at the local level, controlling for individual- and area-level measures. Our findings support a shift in focus away from individual factors and towards altering the landscape of women’s decision making contexts when considering behaviours relevant to public health.

## Teaser

Using breastfeeding initiation and duration and local-level indicators of environmental quality, we test the life history theory prediction that parental investment will be lower in harsh environments. We find that local environmental quality (as rated by an independent assessor) positively predicts breastfeeding over and above the influences of individual SES and the wider environment. Higher SES also appears to buffer against the negative effects of lower local environmental quality.

## BACKGROUND AND OBJECTIVES

### Breastfeeding as maternal investment

The benefits of breastfeeding are well established [1] with benefits for infants (e.g. reduced risks of developing respiratory diseases, gastrointestinal conditions and various other infections[2–4]), mothers (e.g. reduced risk of being overweight and developing diabetes and some female cancers[5–7]) and society (e.g. reduced financial and environmental costs and parents needing less time off to care for sick infants [8]). Despite its many benefits, many women in high-income populations do not breastfeed and of those that do, few manage the WHO recommended 6 months of exclusive breastfeeding [9]. The UK has particularly poor breastfeeding rates [10], inspiring many interventions to improve participation in recent years [11,12]. Breastfeeding is patterned socioeconomically [13], ethnically [14] and geographically [15], with great disparities across the country.

In the UK’s context, whether an infant is breastfed does not represent the same life-and-death situation as it would have done throughout most of human history [10]. However, there are still advantages to receiving breastmilk: reduced hospital admissions [3], better cognitive development [16] and resilience against psychosocial stress[17, 18]. Breastfeeding support groups advocate that every drop of breastmilk counts [19] and there is some truth behind this sentiment, with some benefits of breastfeeding being dose-dependent. For example, reductions in hospital admissions for non-perinatal infections are seen for each additional month of breastfeeding [3]; children exclusively breastfed for as little as 3 months have higher IQ scores than those breastfed for less than 3 months, and scores are higher when breastfeeding is maintained for longer [16]. Even one day of breastfeeding has benefits, with colostrum being particularly valuable for newborns [18, 20]. Breastfeeding initiation and duration are not just relevant to public health [10, 21, 22], but also important indicators of parental investment in offspring quality.

Life history theory emphasises trade-offs in energetic resources across the lifespan, including those surrounding parental investment [23] and thus the framework helps to understand differences in breastfeeding behaviour within populations, and might help to explain the variation seen in the UK. Breastfeeding is energetically costly for mothers, requiring twice as much daily energy as gestation [24]. It is additionally time-consuming and can prevent mothers from engaging in other activities [24, 25]. Like other depreciable forms of parental investment, breastfeeding necessarily affects the amount of resources available for women to invest in their own growth or future reproduction, or caring for other current offspring and assisting other kin [26]. As such, women must make trade-offs regarding the level of investment to provide through lactation [27]. For example, shortened breastfeeding duration may reflect a (conscious or unconscious) decreased investment in the current offspring in favour of being able to reproduce again soon [28, 29], while extended breastfeeding durations may indicate higher investment. This is not to imply a qualitative judgement of women’s parenting decisions, or to say that women who do not breastfeed are investing less in their offspring, but rather to acknowledge breastfeeding as one of several ways in which mothers can invest in their children.

Breastfeeding may not be a very straightforward predictor of parental investment, however, especially in high-income contexts. Women may feed a child formula rather than breastmilk, not through any deliberate reduction of parental investment, but to allow investment in other ways, e.g. economically rather than energetically. It isn’t clear how women weigh up the relative costs of feeding their children. Deciphering this trade-off may be especially complex for well-nourished women where gaining sufficient calories to breastfeed is not a problem.

In our evolutionary past, not breastfeeding an infant would almost certainly result in death. Such complete withdrawal of lactational investment may not have been common, but every woman would have faced decisions about how long to breastfeed for. Given lactational amenorrhoea’s role in preventing subsequent pregnancy [30, 31], reducing or stopping breastfeeding would have been an effective way of reallocating investment, shifting focus from current offspring to future reproduction [32]. Decisions about whether and for how long to breastfeed may therefore have been crucial for allocating maternal investment optimally between children. Such decisions are underpinned by evolved psychological and physiological mechanisms which may still have behavioural consequences in the evolutionarily-rare context of minimal breastfeeding we see in many high-income societies today.

### Life history theory and environmental influences on reproductive strategies

Life history trade-offs are influenced by one’s environment [33], and more specifically ‘environmental quality’. Two key components of ‘environmental quality’ are resource access and extrinsic mortality risk.

Resource availability affects women’s overall energy budget. Individuals with larger budgets are able to invest more in both parental care *and* fertility [33, 34]. Resource access can refer to extra-somatic resources (e.g. income, education and job status), as well as embodied capital gleaned form support networks [35]. Social support [36, 37] may be a particularly important resource in a social, cooperatively breeding species such as ours [38]. Resources can also be somatic, i.e. an individual’s condition, with physiological and psychological quality likely affecting trade-off decisions.

Extrinsic mortality risk, i.e. risk not dependent on an organism’s own behaviour [39], also shapes life history trade-offs: individuals in higher mortality environments are predicted to have relatively early births [40] and more births [41, 42], in order to achieve reproductive success before dying (although see [43] for descriptions of non-linear associations). Lower parental investment per offspring may also be a characteristic of high extrinsic mortality risk [44]—though this is likely confounded by lower resource access.

It is hard to measure extrinsic mortality risk and resource access/scarcity separately, and our analysis cannot disentangle these two components of environmental quality. Our aim instead is to understand environmental influences in more detail by measuring environmental quality at various levels, focusing on localised, subjective experience and exploring the possible distinction between sociocultural and physical aspects of the environment. We use three sets of indicators of both resource access and extrinsic mortality risk: area-level environment, local environment and individual socioeconomic status (SES). We now briefly discuss how environmental quality has been operationalised in other studies before presenting our approach.

### Operationalising environmental quality

Environmental quality is not a concept unique to the life history literature, but is also used in public health, psychology and anthropology to contextualise and explain human behaviour. In high income populations (where most of this research has been done), environmental quality consistently correlates with a wide range of health outcomes and behaviours from chronic diseases and the aging process, to mental health and social well-being [37, 45–49]. It predicts patterns of reproductive behaviours and outcomes, with not just earlier first births and more births, but also preterm deliveries, smaller for gestational age and lower birthweight babies common in poor quality environments [50–55]. Links with parenting strategies have been less well explored [though see 32] but it is likely that breastfeeding, a form of parental investment and important health behaviour, may be similarly amenable to environmental influence [43].

What constitutes a poor quality environment is variably defined, not always well operationalised, and often measured crudely at the aggregate-level. Poorer quality environments can be thought of as having more social and physical environmental problems and less social cohesion [57–59] and as being less safe than higher quality environments [60]. Physical and sociocultural aspects of environmental quality are sometimes conflated [61], but distinctions can help clarify which specific attributes are predictive of different health outcomes [36,62–64]. We conceptualise environmental quality in two main ways: sociocultural environmental quality includes how people in the local area behave towards each other, for example how supportive and friendly people are or whether there are signs of crime and antisocial behaviour; while physical environmental quality captures the built environment as well as notions of cleanliness and pollution.

Environmental quality is often measured as area-level SES, with the Index of Multiple Deprivation (IMD) used most often in UK breastfeeding research. Other measures used include the Child Poverty Index [65] and council tax valuation bands [66]. Although measured at lower spatial scales, the IMD is typically presented as an aggregate measure at the ward-level. IMD studies present mixed results, with higher levels of deprivation linked to earlier breastfeeding cessation in some studies [13, 67] but not others [14]. Nettle *et al.* e.g. found that women in the most deprived neighbourhoods breastfed their infants for almost 3 months less than those living in less deprived areas [52]. These findings support life history theory predictions, but localised measures of environmental quality may better capture an individual's actual experience than crude approximations based on aggregated area-level measures [60, 62].

### The role of subjective environmental experience and environmental perception

There has been a recent shift towards using respondents’ own assessments of environmental quality instead of aggregate-level proxies [49]. As with the more objective measures of environmental quality, individual environmental perception correlates with several reproductive behaviours [62, 68, 69]: women have lower birth weight babies [52, 57] and earlier first births [52, 62] when they *perceive* their environments unfavourably. Parenting strategies are similarly affected; *subjective experience* of mortality (as measured by number of children lost under the age of 15) negatively predicts maternal involvement with offspring [56]. Subjective environmental quality is also more strongly linked to some health outcomes than objective environmental quality [49, 57, 60], but researchers emphasise the need to explore both kinds of measure to gain a more comprehensive understanding of links between the environment, behaviours and health outcomes [57, 61].

### SES as marker of individual condition and a buffer to environmental insults

In contemporary high-income countries, evolutionary researchers need to take into consideration heterogeneity and stratification in the populations they study, especially in large and economically unequal societies such as the UK [70–72]. SES, however, is a biologically problematic construct, more readily explained culturally than with biology [73]. In evolutionary studies, it has been conceptualised as representing an individual’s condition (which may incorporate ‘scarring’ from living in a high extrinsic mortality environment) [74–76] or a marker of the resources a parent has [77]. As such, teasing apart individual and environmental components of mortality risk or resource access becomes tricky. However, individual condition and resource access may influence the trade-offs mothers make regarding how best to invest their resources, over and above environmental factors and vice versa [45, 75, 78]. For example, people in poor communities may experience a ‘double jeopardy’ where socioeconomic stressors interplay with environmental hazards to have negative impacts on health, while those with higher SES are protected against environmental insults by virtue of their greater access to resources and better condition [55, 79–81]. We therefore additionally conceptualise individual SES as a means to buffer against risks posed by low environmental quality—as judged both subjectively and objectively.

### Aims and hypotheses

The overall aim of the study is to investigate whether localised measures of environmental quality are associated with breastfeeding initiation and duration, and to tease apart the influence of local environmental experience and individual SES on women’s investments in breastfeeding in the UK. We will address this by testing two main hypotheses:

1. Local environmental quality is positively correlated with the probability of breastfeeding initiation and lengthened breastfeeding duration;
2. Higher individual SES buffers against negative effects of lower local environmental quality on breastfeeding.

**Figure 1.**
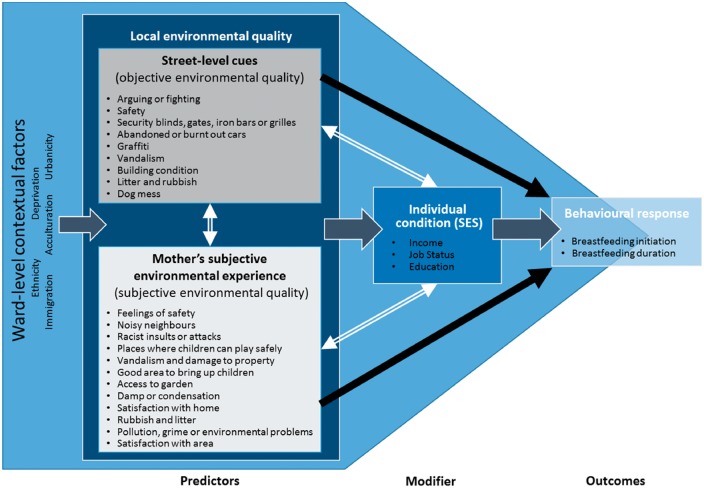
Conceptual framework. This figure shows how we conceptualised relationships between environmental quality, socioeconomic status and breastfeeding outcomes, and how we operationalised them in statistical models. White double-headed arrows represent predicted interactions. Black single-headed arrows represent predicted positive associations. The wide dark grey arrows represent assumed links not explicitly tested in our models. We constructed objective environmental quality and subjective environmental quality scores based on a factor analysis of independent neighbourhood assessments and mother’s survey responses: bullet points show the items each measure is comprised of

In acknowledgement of the potential influence of larger-scale environmental factors, we also consider area-level environmental quality (measured by IMD) and other contextual factors in our models to isolate local level influences on breastfeeding, above and beyond wider-scale deprivation. Our conceptualisation of the layers of environmental influence on women’s breastfeeding behaviours is shown in Fig. 1.

## METHODOLOGY

### Sample

The Millennium Cohort Study (MCS) is an ongoing longitudinal study following the lives of around 19,000 children born in the UK between 2000 and 2002 [82, for a full cohort profile see 83]. We use information collected in the first and second waves, where children were around 9 months and 3 years old, respectively [84, 85]. Geographical boundary data provide larger-scale environmental influences at the ward/superward level [86, 87]. We restricted the sample to biological mothers still living with their children and, where mothers had twins or triplets, we only included data from one child (Cohort Member 1). Samples were further restricted to mothers who completed both waves of data collection. This gave us a maximum usable sample size of 14,576 mothers.

### Variables

#### Outcomes

Breastfeeding initiation was measured retrospectively by asking mothers whether they had ever tried to breastfeed, and duration was captured by asking the age at which the infant had last received breastmilk. Initiation does not therefore confirm breastfeeding success nor is duration necessarily limited to exclusive breastfeeding. Both outcomes were measured in Wave 1 and some mothers were still breastfeeding at the time of this survey.

#### Predictors

We used weighted iterated principal factor analysis with oblique promax rotation to create summary measures of environmental quality [88, 89]. We included 29 items (listed in Table 1) chosen to reflect both physical and sociocultural aspects of the environment: 19 from interviews with mothers; and 10 from neighbourhood assessments.

**Table 1.** Factor analysis results: pattern matrix with rotated factor loadings

Item	Source	Factor 1	Factor 2	Uniqueness	Aspect
		Objective environmental quality	Subjective environmental quality		
Support sought since birth	S1 MAIN	0.1357	-0.0401	0.9854	Socio
Frequency spends time with friends	S1 MAIN	-0.0049	0.0450	0.9982	Socio
Other parents can talk to	S1 MAIN	0.0982	0.1656	0.9469	Socio
Noisy neighbours	S1 MAIN	-0.0594	**0.6999**	0.5477	Socio
Racist insults or attacks	S1 MAIN	-0.0859	**0.7242**	0.5297	Socio
Any places where children can play safely	S1 MAIN	0.1057	**0.3146**	0.8570	Socio
Feelings about neighbour friendliness	S1 MAIN	0.0453	0.2955	0.8974	Socio
Access to garden	S1 MAIN	0.1589	**0.4018**	0.7502	Phys
Central heating in house	S1 MAIN	0.1357	0.1891	0.9204	Phys
Damp or condensation	S1 MAIN	0.1043	**0.3212**	0.8528	Phys
Satisfaction with home	S1 MAIN	0.0471	**0.4885**	0.7364	Phys
Rubbish and litter	S1 MAIN	0.0082	**0.7746**	0.3937	Phys
Vandalism and damage to property	S1 MAIN	−0.0224	**0.7948**	0.3854	Socio/Phys
Poor public transport	S1 MAIN	−0.1250	0.1680	0.9769	Socio/Phys
Food shops in easy access	S1 MAIN	−0.0197	−0.0489	0.9963	Phys
Pollution, grime, environmental problems	S1 MAIN	−0.1056	**0.6491**	0.6353	Phys
Satisfaction with area	S1 MAIN	−0.0074	**0.6986**	0.5170	Socio
How safe feel in area	S2 MAIN	0.2274	**0.3655**	0.7325	Socio
Good area to bring up children^a^	S2 MAIN	**0.3488**	**0.3978**	0.5829	Socio
General condition of buildings on the street	S2 NA	**0.7557**	0.0883	0.3552	Phys
Security blinds etc.	S2 NA	**0.7144**	0.0184	0.4763	Socio
Volume of traffic	S2 NA	0.1498	−0.0034	0.9781	Phys
Burnt out cars on the street	S2 NA	**0.5715**	−0.1485	0.7352	Socio/Phys
Litter etc. in the street or on the pavement	S2 NA	**0.8105**	0.0498	0.3007	Phys
Dog mess on the pavement	S2 NA	**0.7619**	−0.1271	0.4991	Phys
Graffiti on walls or in public spaces	S2 NA	**0.8866**	−0.0682	0.2691	Socio/Phys
Evidence of vandalism	S2 NA	**0.9211**	−0.1428	0.2611	Socio/Phys
Arguing or fighting on the street	S2 NA	−**0.3872**	−0.1154	0.7927	Socio
Observer feeling in the street	S2 NA	**0.7895**	0.1246	0.2639	Socio

Factor loadings greater than 0.3 were included in the main environmental quality measures and are shown in bold. Items loaded on to two factors. Weighted *n* = 16 954. S1 MAIN: mothers' answers to main survey carried out when child was ∼ 9 months old. S2 MAIN: mothers' answers to main survey carried out when child was ∼ 3 years old. S2 NA: second survey neighbourhood observations. Socio: Sociocultural environment. Phys: Physical environment. Cronbach’s alpha coefficients: Factor 1 = 0.80, Factor 2 = 0.81.

a This item loaded onto both factors but was only used in the subjective environmental quality measure as it was reported by the mother not the neighbourhood assessor.

##### Interview items

Mothers were asked questions regarding their local area (‘within about a mile or 20 minutes walk’ [90]) and their home. Seventeen of the 19 items were taken from the first wave and the other two from the second wave. These items provide a balanced spread of an individual’s own environmental experience: focussing on both the immediate environment (the home) and the external broader local environment (the self-defined local area); and include both physical and sociocultural information.

##### Neighbourhood assessment items

Supplementary neighbourhood observations were carried out during Wave 2 of the MCS as part of an evaluation of The National Evaluation of the Children’s Fund [91]. Non-resident observers responded to 11 questions about the general state of the neighbourhood and reported how safe they felt for each visit they made to the household [92, 93]. We included all but one of these measures in our factor analysis (traffic calming excluded due to a high level of missingness at 60.1%). Households were visited on several occasions, with the majority being visited two or three times and some being visited as often as 15 times [92]. We created an average score for each item across all visits to account for any time-based variation. Unlike the interview items relating to a mother’s own perception and experience of her environment, these neighbourhood assessment items reflect a more objective account of the local area. For example, assessors are likely to have calibrated their assessments through exposure to multiple neighbourhoods during the study wave. These neighbourhood observations have been shown to map well on to both how disadvantaged an area is (as defined by the Child Poverty Index) and the criteria used to allocate Children’s Fund programmes [92], lending further support to their use in creating an objective measure of environmental quality.

##### Factor analysis

Prior to analysis, we had expected the interview and neighbourhood assessment items to represent the same underlying construct of local environmental quality with perhaps distinct physical and sociocultural dimensions emerging. However, based on eigenvalues over 1[94], the two factors that were identified could be better considered as relatively more objective and relatively more subjective indicators of environmental quality. Objective items were entirely reported by the neighbourhood assessor and subjective items entirely by the mother. The factor loadings are shown in Table 1.

Only items with factor loadings above 0.3 were included in the measures, resulting in twelve being included in the subjective measure and nine in the objective measure. Whether the mother thought that she lived in a good area to bring up children loaded on to both factors, but we decided to only include it in the subjective measure as this was a response provided by the mother, not the neighbourhood assessor. Eight variables did not load on either factor and we test their relationships with breastfeeding outcomes in separate models (results shown in the supplementary material).

Confirmatory factor analysis was then used to predict objective and subjective environmental quality factor scores. The Cronbach’s alpha coefficient was 0.81 for the subjective and 0.80 for the objective measure indicating good inter-item reliability.

##### SES

As SES can be variably defined and measured, we opted to use three indicators: income, job status and education. We ran separate sets of models for each indicator, and one set of models including all three. Income was equivalised to take account of household composition. Job status was measured by the National Statistics Socio-economic Classification and education by highest qualification level. We combined academic and vocational qualifications into one variable using the information on the government’s education and learning page [95]. For partnered mothers, the higher job status and qualification level of her and her partner was used.

There were some differences between the different SES model versions but none that affected our substantive conclusions. We therefore focus mainly on results from the income models, presenting models using the other indicators in the supplementary material.

## COVARIATES

### Exposure to current environment

We included time at current address and whether women moved house between waves to control for duration of exposure to current environment. We acknowledge that for those who moved house their former environment may have been different from their current environment. On average we might expect the two environments to be relatively similar (with some women moving to higher quality areas, others to lower quality areas and many to areas of similar quality). The vast majority of women would’ve stopped breastfeeding in the interval between Wave 1 (when the child was 9 months old) and Wave 2 (3 years old) and so we largely avoid the issue of using a new environment to predict past behaviour. We ran models with a restricted sample (non-movers only; not shown) but found substantively similar results, with similar-sized effects going in the same direction and with similar levels of significance.

### Infant and maternal characteristics

We included several infant and maternal characteristics known to be important for predicting breastfeeding outcomes: birthweight [96], maternal age [97], partnership status [98], parity [65, 99, 100], ethnicity [101], immigration and acculturation [102].

Maternal age was coded into roughly 10-year age bands. We used number of parents/carers in the household as a proxy for partnership status, although some mothers may be partnered but not cohabiting. We used number of siblings of cohort member in household as our parity measure, although we note that this may underestimate parity for cases where children have left the family home. Ethnicity was coded into four categories due to small sub-group sizes. We chose cohort member ethnicity rather than mother’s ethnicity to capture the combination of maternal and paternal ethnicity-related influences on breastfeeding. Immigration status was derived from respondent’s place of birth and their parent’s place of birth and coded into born in the UK, second generation, first generation (arrived as child) and first generation (arrived as adult) to reflect varying degrees of cultural assimilation. Language(s) other than English spoken at home was used a measure of acculturation. Birthweight was categorised as low, normal or high.

### Contextual factors

We included several contextual factors in our models to isolate local level influences on breastfeeding, above and beyond wider-scale deprivation. Our conceptualisation of the layers of environmental influence on women’s breastfeeding behaviours is shown in Fig. 1. Ward-level IMD scores accounted for larger-scale environmental influences and weighted ward-level proportions of immigrants, speakers of other languages, black and ethnic minorities, and people living in urban areas controlled for geographical sociocultural variation.

Immigration composition was derived by calculating the proportion of women who were born in the UK and whose parents were born in the UK for each ward and using its inverse to calculate the proportion that could be classified as immigrants. For language composition we calculated the proportion of people in each ward that spoke only English and used its inverse to give a proportion of people who either spoke English and another language or just another language at home. Similarly, ethnic composition was created by taking the inverse of the proportion of White mothers by ward. The urban proportion was simply the average number of people living in urban areas by ward and for IMD we used the weighted mean score by ward.

## ANALYSES

We used logistic regression to investigate associations between our two local environmental quality measures and the probability of initiating breastfeeding. For breastfeeding duration, continuous-time event history analyses accounted for the right-censored nature of the data with analyses necessarily restricted to mothers who reported initiating breastfeeding (*n* = 12,182). Time to termination of breastfeeding was measured in months. Based on the shape of the hazards for stopping breastfeeding, we used the Weibull distribution, allowing hazards to increase and decrease smoothly over time [103, p305]. We checked for the suitability of this approach by testing for interactions between all predictor variables and time [103, p307] and checked that the proportional hazards assumption was verified [104, p282].

Mixed-effects models were used for both outcomes to account for the hierarchical structure of the data, with individual mothers (all only included in the analysis once) clustered within set wards/superwards. The random effect for ward/superward accounted for unmeasured variability due to higher-level environmental factors. All analyses were weighted using MCS Wave 2 sample weights to account for the stratified clustered sampling design and drop out between waves [105]. Analyses were conducted in STATA/SE v.14.0 within the UK Data Service’s Secure Lab [106].

To test whether local environmental quality is positively correlated with the probability of breastfeeding initiation and duration (H1), we ran models for each breastfeeding outcome, including each of our environmental quality measures separately, adjusting for (i) maternal and infant characteristics, (ii) SES and (iii) contextual ward-level factors. Given that infant feeding is ultimately an individual decision, we built our model up in this way to test whether individual-level factors remained associated with breastfeeding outcomes once the larger-scale environmental factors had been accounted for. We present results from this fully-adjusted model and show model progression in the supplementary material (SM Table 3 and Supplementary Table S4). To test whether higher SES buffers against negative effects of lower local environmental quality on breastfeeding (H2), we tested for interactions between SES and environmental quality in the fully-adjusted models. We considered there to be evidence of an interaction when the Wald Test *P* ≤ 0.05. Significant interactions are presented graphically.

## RESULTS

### Characteristics of study sample

69.44% of mothers reported initiating breastfeeding and the mean duration was 2.70 months (SD 3.49) (Table 2). The lowest breastfeeding initiation rates and durations were found in women with low subjective (64.84% and 2.35 months) and objective (60.29% and 2.08 months) environmental quality scores. The environmental quality variables that did not load on to the two main measures were generally similarly associated with breastfeeding outcomes, with mothers in poorer quality environments exhibiting reduced breastfeeding behaviour. In terms of ward-level environmental quality, women who did not initiate breastfeeding shared similar characteristics to those who did initiate but had the shortest breastfeeding duration. They were more likely to live in an area with few black and ethnic minority and immigrant inhabitants, and few people who did not speak English; and they were more likely to live in an urban and more-deprived area. Additional descriptive statistics can be found in SM Table 2.

**Table 2.** Descriptives for key variables

		Breastfeeding	
	*n*	Initiation (*n*(%))	Duration in months (Mean (SD))
Environmental quality			
Subjective environmental quality***			
Low	5,038	3,266 (64.84%)	2.35 (3.37)
Middle	4,543	3,192 (70.28%)	2.77 (3.52)
High	4,576	3,347 (73.14%)	2.98 (3.56)
Objective environmental quality***			
Low	5,580	3,360 (60.29%)	2.08 (3.24)
Middle	4,362	3,095 (70.99%)	2.75 (3.51)
High	4,173	3,329 (79.79%)	3.46 (3.63)
Individual condition (SES)			
Income (OECD equivalised quintiles)***			
Lowest	3,271	1,753 (53.61%)	1.67 (3.01)
Second lowest	3,153	1,950 (61.87%)	2.12 (3.26)
Middle	2,815	1,972 (70.05%)	2.69 (3.54)
Second highest	2,742	2,186 (79.72%)	3.34 (3.62)
Highest	2,555	2,230 (87.28%)	4.06 (3.57)
Job status (NS-SEC)***			
Not applicable	1,027	587 (57.38%)	2.04 (3.30)
Routine and manual	4,810	2,647 (55.09%)	1.56 (2.84)
Intermediate	2,775	1,914 (69.00%)	2.50 (3.44)
Higher managerial, administrative, professional	5,964	4,966 (83.28%)	3.82 (3.69)
Education (highest qualification)***			
None	2,396	1,294 (54.05%)	1.79 (3.13)
Level 1 or 2	5,289	3,178 (60.09%)	1.80 (2.98)
Levels 3 to 5 (inc. others and overseas)	3,071	2,262 (73.66%)	2.85 (3.48)
Level 6 plus	3,789	3,365 (88.81%)	4.42 (3.72)
Total^a^		10,114 (69.44%)	2.70 (3.49)

Unweighted. *N* = 14,576. Pearson Chi^2^ comparing proportion initiating breastfeeding across categories:

*** *P* ≤ 0.001. SES: socioeconomic status. OECD: Organisation for Economic Co-operation and Development. NS-SEC: National Statistics Socio-economic Classification.

a Initation data missing for 11 mothers.

### Model results

As the model covariates are all well-established risk factors in the breastfeeding literature, we do not discuss their relationships with the breastfeeding outcomes further here, and return our focus to our localised measures of environmental quality.

### H1. Associations between local environmental quality and breastfeeding outcomes

#### Subjective environmental quality

Subjective environmental quality was positively associated with breastfeeding initiation when controlling for maternal and infant characteristics: a one-point increase in subjective environmental quality predicted 12.5% greater odds of breastfeeding initiation (CI 1.026–1.234). Subjective environmental quality did not however predict breastfeeding initiation once SES and/or ward-level contextual factors were accounted for (Table 3; see Supplementary Table S4 for model progression). Results did not vary according to the SES indicator used (Supplementary Tables S5–7). We also tried adding just IMD (or IMD plus the other ward-level factors) but not SES to the models (results not shown). This also made the relationship between subjective environmental quality and breastfeeding initiation disappear suggesting that both individual and broader-level measures may be better measures of environmental quality than our more localised measure of environmental perception.

**Table 3.** Associations between subjective and objective environmental quality measures and breastfeeding outcomes

	Initiation			Termination		
	Odds Ratio	95% Confidence Interval	*P*-value	Hazard Ratio	95% Confidence Interval	*P*-value
Subjective environmental quality	0.964	0.880-1.057	0.438	0.947	0.896-1.001	***0.056***
Income (OECD equivalised quintiles)			**<0.001**			**<0.001**
Lowest	1.000	(ref.)	.	1.000	(ref.)	.
Second	1.156	0.929–1.437	0.193	0.905	0.791–1.036	0.149
Middle	1.599	1.263–2.024	**<0.001**	0.811	0.712–0.923	**0.002**
Fourth	2.382	1.862–3.046	**<0.001**	0.775	0.682–0.882	**<0.001**
Highest	2.704	1.970–3.710	**<0.001**	0.765	0.673–0.869	**<0.001**
Constant	0.354	0.198–0.632	**<0.001**	1.054	0.759–1.463	0.754
*N*	13,852			9,620		
Objective environmental quality	1.537	1.229–1.922	**<0.001**	0.859	0.766–0.965	**0.010**
Income (OECD equivalised quintiles)			**<0.001**			**0.002**
Lowest	1.000	(ref.)	.	1.000	(ref.)	.
Second	1.072	0.870–1.322	0.513	0.941	0.821–1.078	0.381
Middle	1.411	1.126–1.768	**0.003**	0.844	0.738–0.966	**0.014**
Fourth	2.006	1.570–2.563	**<0.001**	0.800	0.699–0.916	**0.001**
Highest	2.181	1.587–2.999	**<0.001**	0.782	0.685–0.893	**<0.001**
Constant	0.092	0.041–0.206	**<0.001**	1.346	0.898–2.017	0.150
*N*	13,737			9,561		

Each model includes one environmental quality measure only. Models are adjusted for exposure to current environment, infant and maternal characteristics, income and ward-level contextual factors. *P*-values ≤0.05 shown in bold and *P*-values between 0.05 and 0.1 shown in bold italic. Hazard ratios represent breastfeeding termination rather than duration. The number of observations (*N*) varies between models due to differing levels of missing data. Results weighted to allow for complex survey design and models are hierarchical to control for clustering at ward-level. OECD, Organisation for Economic Co-operation and Development.

For breastfeeding duration, hazard ratios are interpreted as the probability of stopping breastfeeding. We found (weak) evidence that higher subjective environmental quality correlated with lengthened breastfeeding duration after controlling for all covariates. A 1-point increase in subjective environmental quality predicted a 5.3% reduction in the odds of termination per month (CI 0.896–1.001). However, we have little confidence in this relationship as the effect size was small and the relationship disappeared in models when alternative SES indicators were used (Table 3 and Supplementary Tables S5–7).

#### Objective environmental quality

Objective environmental quality positively predicted both breastfeeding initiation and duration. In the fully adjusted model, a one-point increase in objective environmental quality predicted 53.7% greater odds of breastfeeding initiation and a 14.1% reduction in the odds of breastfeeding termination per month (Table 3). The effect sizes varied slightly when alternative SES measures were used and only breastfeeding initiation remained significantly associated with objective environmental quality in all three other SES model versions (see Supplementary Tables S5–7). The equivalent estimates ranged from 29.6–53.5% for initiation and 3.7–10.7% for duration. For full model results, including estimates for all control variables and random effects, see Supplementary Table S3.

#### Other environmental quality indicators

Some of the extra environmental quality variables that did not load onto the two main measures had significant associations with breastfeeding outcomes in their own right, with some remaining predictive of breastfeeding outcomes even after controlling for the summary environmental quality measures and across all SES versions (Supplementary Tables S4–7). The items with the strongest evidence for relationships with breastfeeding outcomes were support sought since birth, having other parents to talk to and spending time with friends, with some evidence also suggesting that neighbour friendliness and central heating may also predict breastfeeding. Associations were largely in the predicted directions of environmental quality positively predicting breastfeeding. We found little to no evidence to suggest that public transport, access to food shops and volume of traffic predicted breastfeeding outcomes and so do not consider these results further. We discuss model results for the other five items in more detail in the supplementary material.

### H2. Does individual SES buffer the effects of environmental quality on breastfeeding outcomes?

#### SES interactions with local environmental quality

In fully-adjusted models, the odds of initiating breastfeeding increase with income; women in the highest income quintile have 2.2–2.7 times the odds of initiation compared to those in the lowest quintile (Table 3). Similarly, the hazard of stopping breastfeeding decreases with income, with hazards 77–78% lower for women in the highest income quintile compared to those in the lowest quintile (results for other SES measures shown in Supplementary Tables S5–7).

Subjective environmental quality did not interact with any of the SES indicators to predict breastfeeding initiation. For breastfeeding duration, we found weak evidence for an interaction between subjective environmental quality and income (*P* = 0.068). Higher-income women had relatively high probabilities of maintaining breastfeeding regardless of subjective environmental quality, while women with lower incomes had higher odds of breastfeeding with higher subjective environmental quality scores.

**Figure 2.**
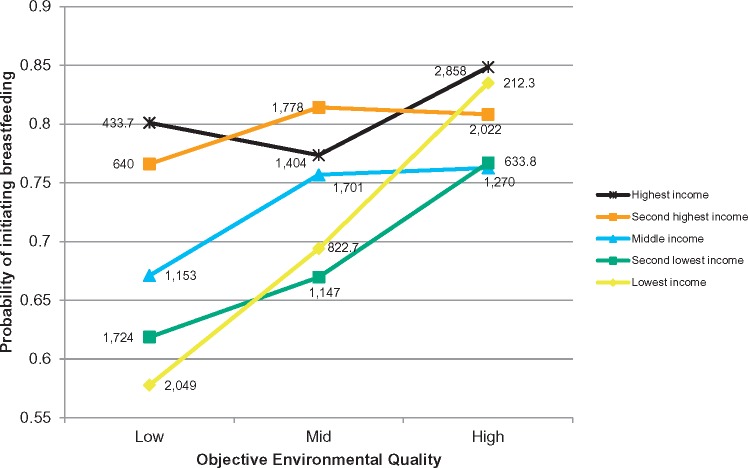
**Breastfeeding initiation by income and objective environmental quality**. Predicted probabilities from model controlling for exposure to current environment, infant and maternal characteristics, income and ward-level contextual factors and accounting for both fixed and random effects. *N* = 13,737. Interaction *P* = 0.013. All categorical covariates held at modal values and continuous covariates held at median values. Data labels are weighted counts for each group

**Figure 3.**
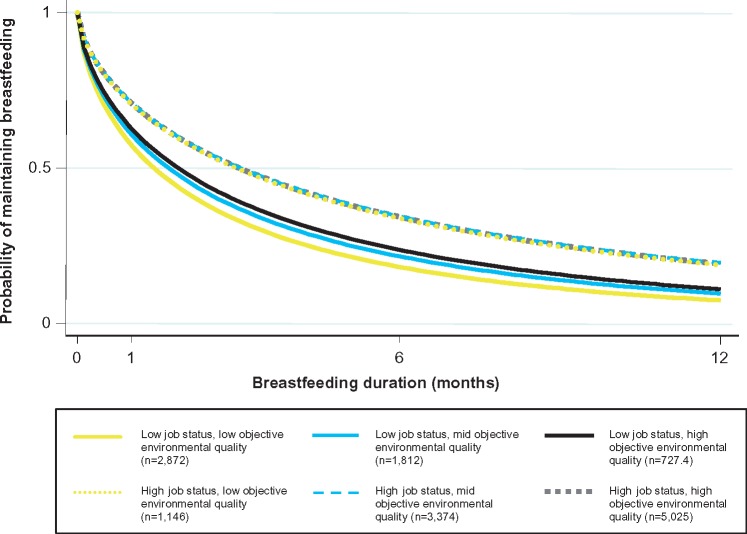
**Breastfeeding duration by job status and objective environmental quality.** Predicted probabilities of breastfeeding duration to 12 months by job status and objective environmental quality. Predicted from model controlling for exposure to current environment, infant and maternal characteristics, income and ward-level contextual factors and accounting for both fixed and random effects. *N* = 9,573. Interaction *P* = 0.045. All covariates held at mean values. Group ns are weighted counts

Objective environmental quality interacted with income to predict breastfeeding initiation (Fig. 2, *P* = 0.013) and with job status to predict breastfeeding duration (Fig. 3, *P* = 0.045), but not other SES indicators. Although we did not find interactions across all SES indicators and for both breastfeeding outcomes, taken together the two interactions provide some evidence that high SES may buffer against environmental insults. Mothers from higher-income households had relatively high breastfeeding initiation rates regardless of objective environmental quality; while breastfeeding initiation was more strongly positively correlated to objective environmental quality in lower-income households. Similarly, mothers from households with high job status were likely to maintain breastfeeding regardless of their objectively-assessed environmental conditions; while the probability of maintaining breastfeeding decreased with lower objective environmental quality scores for women in households with low job status.

## Conclusions and implications

We set out to test whether local environmental quality was associated with breastfeeding and whether individual SES buffers against environmental harshness. We found that local environmental quality did positively predict breastfeeding, but the strength of this association depended on how local environmental quality was measured. We had expected separate measures of local physical and sociocultural quality to emerge from our factor analysis, but these aspects loaded together and items split instead into mother’s own assessments (‘subjective environmental quality’) and those made by an independent enumerator (‘objective environmental quality’). Objective environmental quality was more strongly related to both initiation and duration than subjective environmental quality. We also found some evidence to suggest that individual condition may buffer against environmental insults at the local level.

Our results build on previous life history work which has suggested a link between higher-level environmental quality (as indicated by the IMD) and breastfeeding behaviour (among other life history outcomes) [52]. One of the strengths of our study is that the environment was subjectively defined by mothers and measured on a small scale by neighbourhood assessors. By controlling for contextual factors at the ward level, we were able to see whether smaller-scale local environmental quality and perception had an impact on breastfeeding above and beyond the more distant and already established influences of deprivation, urbanicity, and population composition.

### Comparing environmental quality measures—is environmental perception important?

The ‘objective’ measure of localised environmental quality was a better predictor of breastfeeding outcomes than the ‘subjective’ measure, perhaps surprising as one could expect that mothers’ interview responses would capture actual lived environmental experience better than enumerator assessments [60]. This finding also contradicts the environmental perception literature which suggests that subjective environmental quality has stronger links to health outcomes than objective environmental quality [49, 57, 60].

But objective environmental quality may have stronger associations with breastfeeding than subjective measures because, in this study, it is a better measure of environmental quality. Even though our measures were positively correlated with one another, there was substantial variation in the extent to which the two measures agreed (weighted correlation coefficient = 0.4876), just as agreement between objective and subjective measures in other environmental quality studies has been found to be only low to moderate [61]. We note that the subjective measure was significantly positively correlated with breastfeeding outcomes, but only in models excluding individual-level SES and ward-level factors. Individual SES and broader area-level environmental quality may therefore be more salient predictors of breastfeeding than subjective measures; whereas objective measures of the local environment capture something about environmental quality that is not included in individual or area-level measures. This may be because our two measures are better thought of as capturing *perceived* stressors versus *observed* stressors [36]. Direct measures such as the neighbourhood observations used in our study may capture environmental conditions that are not perceived by residents [61], either because residents have fewer points of comparison than objective observers, and/or because familiarity with an environment affects one’s perception of that environment (making poor quality environments less intimidating for example). Further, mother’s assessments are also likely prone to recall and social desirability bias.

Alternatively, the construction of the measures may provide an explanation for the differences between their associations with breastfeeding. We were restricted by the available variables in the MCS dataset and the subjective measure may have better represented individual exposure to environmental risk if we had had more data on perceptions of problems, cohesion and safety (three dimensions that may be particularly important for determining health outcomes [60]). It would have also been useful to have more information on exposure to crime [57, 107]. Additionally, it may have been illuminating to include a measure of controllability of environmental stressors [108] to try and tease out extrinsic and intrinsic risk. Despite its limitations, the subjective measure was based on more items than the more objective measure and it also had slightly greater inter-item reliability (with a Cronbach’s alpha coefficient of 0.81 vs. 0.80).

Finally, it is also possible that some environmental factors are not particularly salient, and thus not captured by our measure of subjective environmental quality, but may trigger changes in behaviour anyway. This would imply that active environmental perception is not required in order to calibrate reproductive behaviour. We offer stress as a potential mechanism linking environmental quality and breastfeeding.

### Stress as a potential mechanism linking environmental quality to breastfeeding

Mothers in lower quality environments may be more likely to experience psychological and/or physiological stress which in turn may impact their ability to breastfeed. Breastfeeding is an intense commitment and requires frequent nursing to be maintained. Having to deal with environmental problems may make mothers less responsive to their infants as their attention is needed elsewhere. Effort spent trying to remedy problematic environmental situations will necessarily deplete finite physiological resources and the mental capacity needed to persevere with breastfeeding.

Rickard et al. provide oxidative stress-related effects on somatic function as an example of how a stressful environment can translate into a depleted internal state [75]. Our weaker subjective measure associations could suggest that environmental information may be embodied through a means other than perception, i.e. women may not have noticed that their streets were dirty or that there was a lot of vandalism, e.g. but their bodies may still have displayed a stress-response all the same. Similarly, pollution may cause damage to the body without the mind being aware that there are any health-impacting molecules in the air.

The possibility of environmentally induced hormonal and physiological disruption may seem unlikely given the relative stability of the hormonal cascade that results in milk production [109, p89]. However, stress as measured by maternal self-reported exhaustion and stress hormone levels after labour has been found to be associated with the delayed onset of lactogenesis [110] and so the leap from acute stress affecting lactation to chronic stress (i.e. that indicated by poor environmental quality) affecting lactation is perhaps not such a big one. In fact, stress as is manifested by tense, anxious mothers can contribute to the negative cycle of low milk supply and low infant intake. Furthermore, both sociocultural and physical environmental factors have been linked to both a reluctance to breastfeed and a physiological impediment to maintaining and sustaining lactation [109, p361, 111,112].

### The importance of individual condition

Maternal condition and maternal access to resources are important as they influence the trade-offs mothers make regarding how best to invest their energy, including how much to invest in any given offspring. The confounding effect of individual SES on the positive relationship between local environmental quality and breastfeeding is hardly surprising given the well-established socioeconomic differential in breastfeeding in the UK[113]. Although most of the different SES model versions produced comparable results, the fact that some results differed depending on whether we controlled for income, job status, education, or all three SES indicators, supports the notion that these separate elements may reflect different resources a mother has available.

The robust SES-breastfeeding association we observed could be explained in terms of the internal prediction model proposed by Rickard *et al.* [75]. This model suggests that early exposure to psychosocial stress embodies as negative influences on state, which increases morbidity and mortality in adulthood, which in turn calibrates maturation rate. Preparing the body physiologically for breastfeeding may be one component of maturation that can be affected by both current and past environmental exposure influences on internal state.

Sensitivity to environmental conditions is also likely to vary across individuals. Experimental evidence suggests that differential susceptibility may well be patterned by SES [79, 80], with people from low SES backgrounds being more reactive to mortality primes than people from high SES backgrounds [114]. This chimes with the interactions we found between SES and local environmental quality. We predicted that SES would serve as a buffer against environmental insults, modifying the association between local environmental quality and breastfeeding in harsh environments. Our results supported this to some extent because we found that income and job status interacted with objective environmental quality to predict breastfeeding initiation and duration respectively. A lack of social and economic resources may make mothers especially vulnerable as they are not able to easily compensate for what is missing in their immediate surroundings [64].

### Breastfeeding barriers at multiple levels

We focussed on individual and local-level indicators of environmental quality in our analyses and controlled for larger-scale environmental factors to test whether neighbourhood quality and individual experience of the environment can calibrate breastfeeding behaviour. We felt that local-level measures would be more salient than abstract concepts of environmental quality measured in aggregate at higher levels—and thus that they would more accurately capture the cues that women actually process and which trigger behavioural responses. Higher-level environment-breastfeeding links are still likely [13, 52], but our results provide some evidence that local environmental quality predicts breastfeeding outcomes above and beyond the effects of the wider environment. We believe that there will be both breastfeeding-specific aspects (private, welcoming spaces) and more general attributes (cleanliness, friendliness) of both the local and area-level environment that will influence women’s breastfeeding behaviour.

While one of the strengths of this paper is how thoroughly we have investigated environmental quality, there are limitations to our approach. Our two main measures of local environmental quality captured the multiplicity of local environmental experience; mothers do not experience cues in isolation, but rather are exposed to a whole suite of environmental characteristics which are likely to jointly affect individual experience. However, by creating a measure that pools different aspects of environmental quality together, we cannot fully identify which specific aspects of the local environment should be targeted for improvement in interventions; identifying particularly salient and/or influential cues to women’s breastfeeding decisions would benefit intervention development. We explored this to some extent by looking separately at the eight items that did not load on to our two summary measures. While we did not find evidence for effects of the physical environment in these supplementary analyses, we did find some evidence for independent effects of the sociocultural environment on breastfeeding outcomes. Seeking support, having other parents to talk to and spending time with friends were all independently strongly associated (although not all positively) with breastfeeding outcomes, suggesting that these specific aspects of the sociocultural environment can influence infant feeding decisions without necessarily acting in concert with other aspects of local environmental quality. It could be that these particular aspects of environmental experience have more direct influences on breastfeeding, with for example mothers seeking support, or talking to friends and other parents *specifically* about infant feeding—while our summary measures instead represent broader (non-breastfeeding specific) barriers. Further work is needed to tease specific environmental influences apart as there may be little merit in providing a breastfeeding intervention in a neighbourhood where women will not use it because of other environmental problems.

## IMPLICATIONS

With infant feeding back on the political agenda as a result of the recent Lancet breastfeeding series [10, 22], tackling the many barriers that prevent women from breastfeeding has become a priority. Recently, efforts to improve breastfeeding outcomes have shifted focus from individual women to larger societal issues [21]. Evolutionary theory adds value by generating precise predictions and new lines of enquiry that may be missed elsewhere. The findings that emerge from such evolutionary studies are also important for policy makers as they may highlight aspects that policy makers can actually change. The environment can be modified to improve health outcomes with less onus on the individual [28] and is therefore a useful avenue for improving breastfeeding. Our study has shown that there may be broader environmental barriers (environmental quality) behind the breastfeeding-specific social, cultural, economic, physical and practical barriers highlighted by UNICEF [115].

Furthermore, by focusing on differences in environmental quality we can draw attention towards core economic inequities and concentrate on the benefits to be yielded through structural change [116]. There is a historical tradition of placing blame on the individual when he/she becomes sick and the medicalisation of breastfeeding [117] has exacerbated feelings of pressure and guilt for new mothers [118–120]. Breastfeeding is a particularly emotive process with women's sense of self-worth and value intrinsically linked to its success [121, 122]. As such, a shift from the individual towards the environment in infant feeding discourse, and indeed in breastfeeding interventions, would be helpful in improving the emotional wellbeing of mothers and in turn the health of their children. Improving the local environment will undoubtedly have knock-on positive consequences for the health of the rest of the neighbourhood too.

## DECLARATIONS OF FUNDING AND CONFLICTS OF INTEREST

The Millennium Cohort Study is funded by the Economic and Social Research Council (ESRC) and a consortium of government funders. L J Brown is funded by an ESRC Doctoral Training Scheme via the Bloomsbury Doctoral Training Centre, London.

## Supplementary Material

Supplementary Figures

Supplementary Tables

Supplementary Text
