# Supplementary material for: Local environmental quality positively predicts breastfeeding in the UK’s Millennium Cohort Study
Source: Evol Med Public Health. 2017 Aug 21;2017(1):120–35. doi: 10.1093/emph/eox011 (PMC5766197; doi:10.1093/emph/eox011)
Supplement: Supplementary Figures [file eox011_supp_figures.docx]

# Supplementary Figures

Supplementary Figure 1: Breastfeeding duration by subjective environmental quality and objective environmental quality.

Predicted probabilities from model controlling for exposure to current environment, infant and maternal characteristics, income and ward-level contextual factors and accounting for both fixed and random effects. N=9,321. Interaction p=0.003. All covariates held at mean values. Group ns are weighted counts.

**Breastfeeding duration (months)**

0

0.5

1

0

1

6

12

**Probability of maintaining breastfeeding**

Supplementary Figure 2: Breastfeeding duration by job status and how frequently mother spent time with friends in the last week.

Predicted from model controlling for infant and maternal characteristics, job status and ward-level contextual factors. N=9,880. Interaction p<0.001. Those with N/A and mid job status omitted for clarity. All covariates held at mean values. Group ns are weighted counts.

**Breastfeeding duration (months)**

**Probability of maintaining breastfeeding**

0

0.5

1

0

1

6

12
