# Supplementary material for: Local environmental quality positively predicts breastfeeding in the UK’s Millennium Cohort Study
Source: Evol Med Public Health. 2017 Aug 21;2017(1):120–35. doi: 10.1093/emph/eox011 (PMC5766197; doi:10.1093/emph/eox011)
Supplement: Supplementary Text [file eox011_supp_text.docx]

# Supplementary Material

## Interactions between objective and subjective measures of local environmental quality to predict breastfeeding outcomes

When both objective and subjective measures were included in the same model, the positive relationship between objective environmental quality and breastfeeding initiation increased (1.717, CI 1.346-2.191). Conversely, subjective environmental quality became more strongly negatively associated with breastfeeding initiation once we controlled for objective environmental quality, and the relationship became significant (0.872, CI 0.786-0.968). These patterns were replicated in the other SES model versions. Despite this, we did not find any evidence for an interaction between the two measures in predicting initiation.

Subjective and objective environmental quality did however interact to predict breastfeeding duration. As SM Figure 1 shows, most women initiated breastfeeding but only kept it up for a short while, with the biggest difference between groups emerging after 1 month, and relative differences persisting through to 12 months. Women scoring poorly on both measures had the lowest chances of maintaining breastfeeding. Women in objectively measured mid-quality environments with low subjective environmental quality had the greatest chances of maintaining breastfeeding. Gaps between lines show that subjective environmental quality makes a larger difference to the probability of maintaining breastfeeding in low objective environmental quality areas (yellow lines more spaced out) compared to high objective environmental quality areas (black lines closer together). This interaction persisted in all SES model versions (results not shown).

## Results from our models using the extra environmental quality indicators that did not load on to out two main summary measures

### Support sought

We found evidence of a positive association between seeking support and breastfeeding initiation. Mothers who did not seek support had around three quarters the odds of initiating breastfeeding compared to those who did, even after ward-level contextual factors were accounted for. Although the effects sizes varied depending on which indicator of SES was controlled for (SM Tables 4-6 and 11), all results were in the in the direction we predicted: mothers who did not seek support, i.e. those who had a lower quality sociocultural environment, were less likely to initiate breastfeeding. We found no evidence for an association between support sought and breastfeeding duration.

### Other parents to talk to

We found no evidence of an association between having other parents to talk to and breastfeeding initiation in any of the SES models, but we did find evidence for a positive association between having other parents to talk to and breastfeeding duration. After adjusting for SES and contextual factors, those who disagreed or neither agreed or disagreed that they had other parents to talk to had similar hazards of stopping breastfeeding to each other, but those who agreed had a 15.3% reduction in the odds of termination (SM Table 3). The effect size varied slightly across SES model versions with a low of 13.9% for the education and all SES versions (SM Tables 5 & 6) but remained in the direction we predicted: mothers with no other parents to talk to, i.e. with a lower quality sociocultural environment, were less likely to maintain breastfeeding.

### Spending time with friends

We found good evidence of a negative association between how often women spent time with their friends in the past week and whether they initiated breastfeeding. There was no difference in odds of initiation across women who spent between no time and six of the last seven days with their friends, the association was instead driven by those who saw their friends every day in the last week. These women were around 21-23% less likely to initiate breastfeeding compared to those who saw their friends 1-2 times that week (SM Tables 3-6). This goes against our prediction that women with a better quality sociocultural environment are more likely to breastfeed.

How frequently mothers spent time with their friends was even more strongly predictive of breastfeeding duration, but here the association was positive and was driven by the 3-6 times per week group. Those who had no friends or never saw them and those who saw their friends everyday had similar risks of stopping breastfeeding to those who saw their friends 1 or 2 times a week, while those who saw their friends 3-6 times were around 16-17% less likely to stop breastfeeding in the fully adjusted model. This relationship was again robust across all SES model versions (SM Tables 3-6). This is more in line with what we would predict - assuming that spending more time with friends is indicative of having a better quality sociocultural environment.

### Neighbour friendliness

We found no evidence to suggest that feelings about neighbour friendliness predicted breastfeeding initiation. However, we did find some weak evidence for a positive association with breastfeeding duration, although p-values increased above conventional levels of statistical significance once SES was added to the model. This association was driven by the decreased hazard of breastfeeding termination for those who rated their neighbours as friendly, as both those who didn’t express a judgement and those who rated their neighbours as unfriendly did not differ from those who rated their neighbours as neither friendly nor unfriendly. The relationship was in the direction predicted with mothers who had a better quality sociocultural environment (i.e. friendly neighbours) being more likely to breastfeed for longer. The weak evidence of an association found in the other SES model versions does however call the robustness of this relationship into question.

### Central heating

Whether a mother had central heating was positively associated with breastfeeding initiation in the simplest model, but the association was removed once SES was controlled for. Central heating did however predict duration, but not in the predicted direction (and only once SES was added to the models), with mothers being 13-14% less likely to stop breastfeeding when they had no central heating.

## SES interactions with the extra local environmental quality measures

Spending time with friends showed the most pronounced interaction with SES. This variable interacted with all three SES measures. SM Figure 2 depicts how the relationship between how often mothers spend time with their friends and breastfeeding duration varies according to job status. Comparing distances between solid and dashed lines, we can see that high SES is less able to mitigate against reduced breastfeeding chances for women who see their friends less often than for women who see their friends regularly. This goes against our hypothesis that high SES will buffer against low environmental quality, as SES appears to have a stronger impact in a situation we would deem indicative of high sociocultural environmental quality (i.e. high level of support from friends).

## Ward-level variance

As is to be expected, the proportion of total variance due to differences between wards reduced as more variables were added to the models, with the biggest reduction seen between the model adjusting for exposure to current environment, infant and maternal characteristics and SES and the model further adjusting for ward-level factors. Across all sets of models, after accounting for ward-level contextual factors 5.75% of the total variance in breastfeeding initiation was due to unmeasured differences between wards (range 5.31 to 6.31%), while this figure was 1.44% for breastfeeding duration (range 1.33 to 1.48%).
